# Effects of Photoperiod Extension on Clock Gene and Neuropeptide RNA Expression in the SCN of the Soay Sheep

**DOI:** 10.1371/journal.pone.0159201

**Published:** 2016-07-26

**Authors:** Hugues Dardente, Cathy A. Wyse, Gerald A. Lincoln, Gabriela C. Wagner, David G. Hazlerigg

**Affiliations:** 1 PRC, INRA, CNRS, IFCE, Université de Tours, 37380, Nouzilly, France; 2 Institute of Biological and Environmental Sciences, Zoology Building, Tillydrone Avenue, University of Aberdeen, Aberdeen, AB24 2TZ, United Kingdom; 3 Veterinary school, Bearsden Road, Glasgow, G61 1QH, United Kingdom; 4 Queen’s Medical Research Institute, University of Edinburgh, Edinburgh, EH16 4SB, United Kingdom; 5 Department of Arctic and Marine Biology, Faculty of BioSciences, Fisheries and Economy, University of Tromsø, 9037, Tromsø, Norway; Kent State University, UNITED STATES

## Abstract

In mammals, changing daylength (photoperiod) is the main synchronizer of seasonal functions. The photoperiodic information is transmitted through the retino-hypothalamic tract to the suprachiasmatic nuclei (SCN), site of the master circadian clock. To investigate effects of day length change on the sheep SCN, we used *in-situ* hybridization to assess the daily temporal organization of expression of circadian clock genes (*Per1*, *Per2*, *Bmal1* and *Fbxl21*) and neuropeptides (*Vip*, *Grp* and *Avp*) in animals acclimated to a short photoperiod (SP; 8h of light) and at 3 or 15 days following transfer to a long photoperiod (LP3, LP15, respectively; 16h of light), achieved by an acute 8-h delay of lights off. We found that waveforms of SCN gene expression conformed to those previously seen in LP acclimated animals within 3 days of transfer to LP. Mean levels of expression for *Per1-2* and *Fbxl21* were nearly 2-fold higher in the LP15 than in the SP group. The expression of *Vip* was arrhythmic and unaffected by photoperiod, while, in contrast to rodents, *Grp* expression was not detectable within the sheep SCN. Expression of the circadian output gene *Avp* cycled robustly in all photoperiod groups with no detectable change in phasing. Overall these data suggest that synchronizing effects of light on SCN circadian organisation proceed similarly in ungulates and in rodents, despite differences in neuropeptide gene expression.

## Introduction

Organisms living at temperate latitudes experience profound annual changes in their environment (e.g. temperature, food availability), which exert a strong selective pressure. Therefore, animals have evolved endogenous clocks that allow them to adapt to and, most importantly, anticipate those cyclic changes. Photoperiod, the length of the daily light phase, constitutes the most salient and reliable physical cue and is therefore a major synchronizer of physiology [1,2]. In mammals, the retina is the sole photoreceptive tissue and relays photoperiodic information to the main central circadian clock located in the suprachiasmatic nuclei (SCN) of the hypothalamus.

At the molecular level, circadian clocks revolve around a set of clock genes, which interact to cyclically activate and inhibit their own expression. Specifically, a heterodimer of transcription factors composed of CLOCK and BMAL1 turns on the expression of *Period 1–3* and *Cryptochrome 1–2* whose proteins then feed-back onto the dimer to switch off transcription. The CLOCK/BMAL1 dimer also directs transcription of genes that do not directly function in the core clock mechanism but add robustness or instill rhythmicity to various intracellular pathways and higher integrative physiological functions [3,4]. Within the SCN, *Arginine Vasopressin* (AVP) is a well-known example of a clock-controlled gene [5]: AVP can act on distant targets through diffusion in the cerebrospinal fluid and synaptically within adjacent regions, such as the paraventricular nuclei, to which the SCN project [6].

In rodents, each of the two bilateral SCN can be considered to comprise two sub-compartments, a ventro-lateral (core) and a dorso-medial (shell). The neuropeptides *Vasoactive Intestinal Peptide* (VIP) and *Gastrin-Releasing Peptide* (GRP) are characteristic of the core while the shell mostly produces AVP. These sub-compartments show functional dichotomy with the core being the main retino-recipient area while the shell is not directly responsive to light and gets photic information from the core (e.g. [7,8,9]). Furthermore, core and shell project to different nuclei, which are, however, mostly localized within the hypothalamus for both compartments (e.g. [7,8,9]). Induction of *Per1-2* genes by a light pulse at night is therefore not homogenous within the SCN and primarily occurs in the core [10–14]. In contrast to the limited projections to extra-SCN regions arising from the core, there is a dense plexus of VIP and GRP fibers within the rodent SCN (e.g. [9]). These observations have led to an integrated model for photic entrainment: light information induces *Per1-2* gene expression in the core SCN, leading to the secretion of VIP/GRP, which in turn transmit the information to the shell whose clock, and outputs, are progressively reset [15,16].

This model implies that core and shell constitute two distinctly functional units that are nevertheless synchronized *in vivo*, a claim supported by numerous studies [17,18–21]. This working model is also backed-up by other lines of evidence. First, both VIP and GRP can evoke phase-shifts in electrical activity in SCN slices *in vitro*, with response curves similar to those observed with light stimulation [22,23]. Second, lack of VIP signalling caused by gene-targeted disruption of either *Vip* or its cognate receptor *Vpac2* leads to an array of disturbed circadian rhythms that range from low amplitude associated with short period length to arrhythmia in the mouse [24–26]. This might result from aberrant gating of light information [27], lending further support for a role of *Vip* in photic entrainment. Since GRP application can restore rhythmicity in VPAC2 knock-out mice [28,29], there appears to be functional redundancy at least in rodents. Third, the profiles of multiple clock genes in the SCN are differentially affected by photoperiod [30,31]. There are also differences in clock gene expression along the rostro-caudal axis of the SCN, pointing to another level of network complexity [32,33].

The rate at which the phase of the SCN clock resets in response to light is important since it conditions the resetting speed of other body clocks, a hierarchical organization responsible for normal control of circadian physiology and behaviour. Rapid shifts in photoperiodic phasing have cascading effects on this circadian organisation starting with effects on SCN function and then extending to the peripheral body clocks. Transient periods of desynchrony within this cascade are believed to account for most of the inconveniences experienced during jet-lag or shift-work [34,35]. In nocturnal rodent models progressive resynchronisation following shifts in photoperiod have been reported both at the level of the SCN and the level of phasing between different peripheral organs and this master pacemaker [4,15,16].

Data on SCN organisation and light resetting are much more limited for other mammalian groups. In the sheep (the most studied ungulate species), rhythmic patterns of clock gene expression following acclimation to different photoperiods are similar to those seen in rodents [36]. Contrastingly the boundaries between shell and core regions of neuropeptide expression are much less sharply defined than in rodents [37], and although both *Avp* and *Vip* are expressed within the sheep SCN [37,38] their temporal patterns of expression have not been reported.

To determine whether comparative differences in organisation are associated with differences in photoperiodic responsiveness, we compared SCN gene expression in Soay sheep subjected to an acute 8-h delay in lights off. Previously, we have used this photoperiodic switch protocol to explore seasonal neuroendocrine responses, and have assayed it effects on locomotor activity, melatonin secretion and *pars tuberalis* gene expression rhythms [39–41]. These studies demonstrate that while melatonin profiles shift almost immediately, reflecting a strong masking component [40], locomotor activity and *pars tuberalis* gene expression resynchronises in 1 to 2 weeks following the light switch [40,41]. In the present study, our endpoints were daily expression profiles of the clock genes *Per1-2*, *Bmal1* and *Fbxl21* and the neuropeptides *Avp*, *Vip* and *Grp*. Our study reveals that mRNA profiles for *Per1-2* genes respond rapidly over a 15 day period following this photoperiodic switch, paralleling previously described effects on behavioural shifting. In contrast, no effect of the photoperiodic switch was observed on *Vip* and *Avp* RNA expression profiles, while *Grp* was not detected in the sheep SCN. We conclude that SCN-level photoperiodic acclimation proceeds similarly in ungulates and rodents.

## Materials and Methods

### Cloning & constructs

RNA extraction was performed using Tri-reagent (Sigma) according to the manufacturer’s protocol, and cDNA synthesis was carried out using a reverse transcription kit (Qiagen). PCR was done with Platinum Taq Hifi (Invitrogen) according to the manufacturer’s protocol. Constructs used to prepare templates for in-situ hybridization were generated as follows: following agarose gel electrophoresis, PCR fragments of the expected sizes were extracted using a gel extraction kit (Qiagen) and cloned in pGEM-T easy vector (Promega); four to six positive clones were sequenced (MWG, United Kingdom), and the sequences deposited in GenBank (see below).

### Animals

The animals used for this study are the same as those of a previously published paper [39]. Animal experiments were conducted in accordance with the UK Animals (Scientific Procedures) act of 1986 and approved by University of Edinburgh Local Ethics Committee. Fifty-four Soay ewes were brought indoors in January and acclimatised to a 8:16 h light ⁄ dark cycle (SP) for 6 weeks. A first group of 18 (SP group) was killed throughout the day at four hour intervals (n = 3 per time point) starting at ZT0 (lights on) while the remaining 36 animals were switched to a 16:8 h light ⁄ dark cycle (LP) by an 8-h extension of the light phase commencing 8 h after lights on. The day on which the photoperiodic switch was applied was designated ‘day 0’. Animals were then culled as described for the SP condition (every 4h, n = 3 per time point) on days +3 (LP3 group) and +15 (LP15 group) relative to day 0. All animals were killed by an overdose of barbiturate (Euthatal; Rhone Merieux, Essex, UK) administered intravenously. For animals killed in the dark, including those at ZT0 dim red light was used and eyes were covered with opaque plastic sheet until the head had been removed for brain dissection. Hypothalami with pituitary attached were frozen in isopentane cooled over dry ice prior to storage at -80°C.

### *In-situ* hybridisation and quantification of signal

Hypothalamic blocks for *in situ* hybridisation were cut into 20 μm sections using a cryostat, and thaw-mounted onto polylysine and gelatin coated slides. Radioactive cRNA riboprobes were prepared by plasmid linearisation and *in vitro* transcription reactions including ^35^S-UTP (Perkin-Elmer). Sections were hybridized overnight at 60°C with 5 x 10^5^ cpm of probe per slide, subjected to Rnase-A digestion and stringency washes in sodium citrate buffer to remove non-specific probe hybridisation. Slides were then dehydrated in graded ethanol solutions and exposed to an autoradiographic film (Kodak). Exposure duration was optimized for each gene by repeat film exposures, depending on labeling intensity. Films were scanned on a 1640XL transmittance scanner (EpsonUK, Hemel Hempstead, Hertfordshire, UK) along with a calibrated optical density (OD) transmission step wedge (Stouffer, USA). Calibrated Integrated OD measurements of gene expression in the SCN were performed using ImageJ software. Homologous probes used in this paper correspond to the following nucleotide position of GenBank accession numbers: *Avp* nt 1–501 of NM_001126341, *Vip* nt 1–513 of NM_001126368, *Grp* nt 48–545 of NM_001009321, *Per1* nt 1–456 of EF683160, *Per2* nt 1–451 of EF583558, *Bmal1* nt 1–836 of NM_001129734, *Fbxl21* nt 342–709 of NM_001129738.

### Statistical analysis

Data (mean ± SEM) are plotted with respect to zeitgeber time (ZT) where ZT0 = lights on. Data were analysed by ANOVA to test for main effects of photoperiod and ZT as well as photoperiod x ZT interaction. Post-hoc comparisons were made using Tukey test, conducted using GraphPad Prism 6 (La Jolla, USA) with a significance threshold of p < 0.05. Where ANOVA revealed significant effects of ZT, phase was estimated by calculation of circular centre of gravity (CoG) and circular standard deviation using CircWave (http://www.euclock.org/results/item/circ-wave.html).

## Results

### SCN expression of circadian clock genes

**Fig 1:**
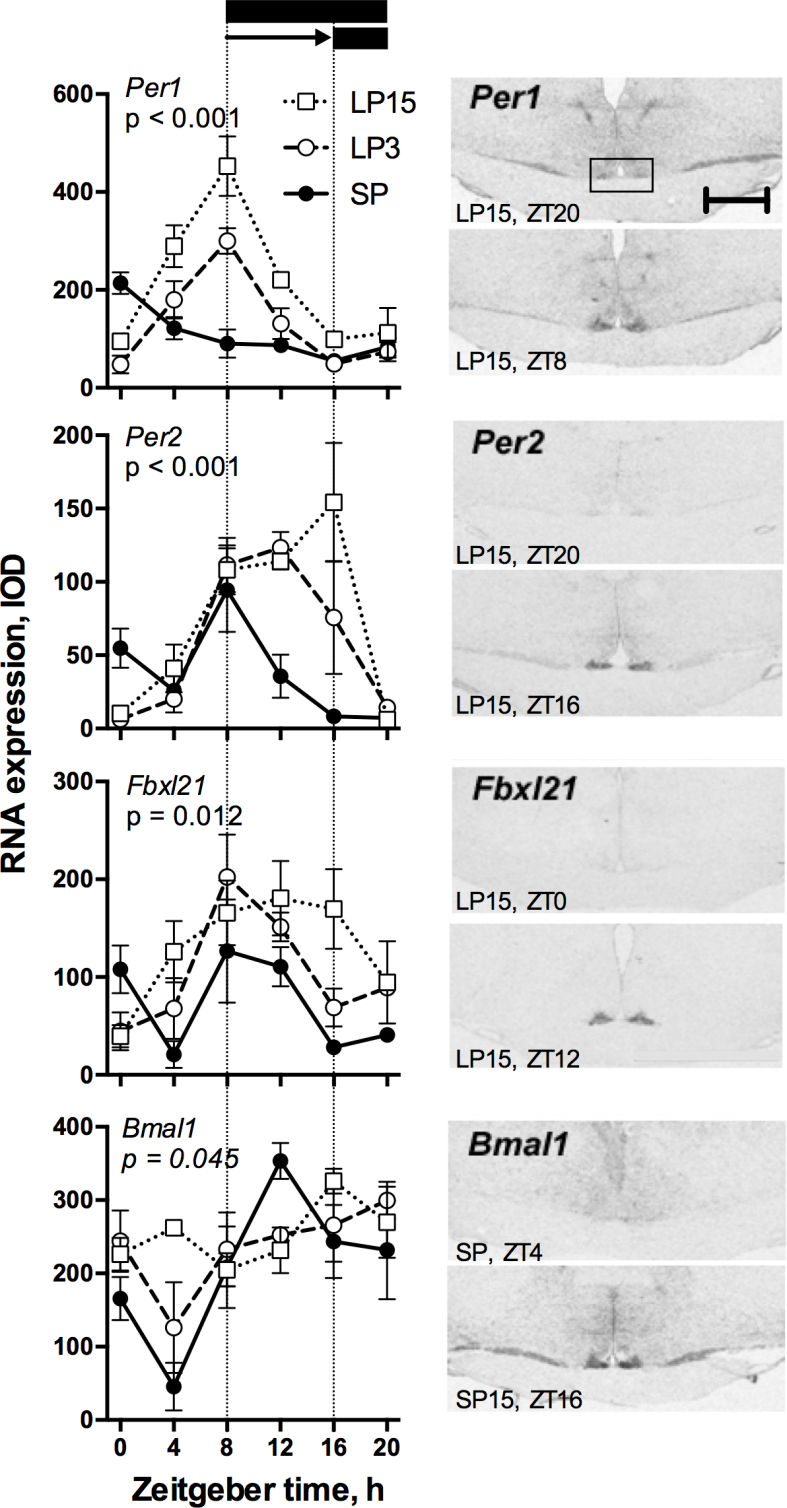
Effects of an 8-h delay in lights off on clock gene expression in the sheep SCN. Left panels show graphs of densitometric analysis for each of the four genes investigated, arranged in order of estimated time of peak expression (*Per1*<*Per2*<*Fbxl21*<*Bmal1*). Data are mean ± SEM of n = 3 animals / ZT point. p values in each graph refer to the F statistic for photoperiod x ZT interaction. The solid horizontal bars indicate the period of the dark phase in SP control animals, and in LP animals following the delay in lights off (horizontal arrow). Right panels show images of minimal and maximal expression for each gene, with phases from which these images were taken. Scale bar = 4 mm.

SCN labelling for the clock genes *Per1*, *Per2*, *Fbxl21* and *Bmal1* is shown in Fig 1.

Prior to the photoperiodic switch, the SCN expression rhythms of the clock genes *Per1* and *Per2* were similar to those described previously in SP acclimated sheep [36], peaking in the early and late light phase respectively (p < 0.001 for overall effect of ZT). Moreover, the expression profiles of each of these three genes changed significantly in the 15 days following the PP switch (p values for photoperiod x ZT interaction: *Per1*/*Per2* –p < 0.0001, *Fxbl21* –p = 0.012).

Under SP, *Per1* expression was maximal during the early day (CoG = ZT1.47 ± 2.83), after which levels steadily decreased to reach a nadir during the mid-dark phase (ZT16). The photoperiodic switch led to clear delay in phasing relative to lights on within 3 days, with maximal levels observed at ZT8 (CoG = ZT7.53 ± 2.18) and a trough during the mid-dark phase, similar to the SP condition. There was no further shift in phasing after 15 days (CoG = ZT7.57 ± 2.31). In addition, compared to the SP condition, daily mean *Per1* levels were increased by about 20% at LP3 and nearly doubled at LP15 (p < 0.001 for overall effect of photoperiod). This difference was attributable to maximal recorded levels of expression increasing under LP, so that by LP15 they were more than 2-fold higher than in SP animals, and to an increased duration of elevated expression relative to the night-time nadir. The ZT20 nadir was not significantly different for the 3 photoperiod groups.

Under SP, the highest *Per2* mRNA levels were observed at ZT8 (CoG = ZT6.65 ± 2.26). The LP transfer broadened the peak of expression with maximal levels observed at ZT8-ZT12 in the LP3 group (CoG ZT11.3 ± 1.68) and ZT12-ZT16 in the LP15 group (CoG ZT12.17 ± 1.96), thereby completing an approximately 6-h phase delay in peak levels. Similar to *Per1*, daily mean levels of *Per2* mRNA in LP3 and LP15 groups were enhanced by ~60% and ~100% compared to the SP group (p < 0.001 for overall effect of photoperiod), and again this was due to higher peak levels of expression and a longer period of elevated expression relative to invariant ZT20 nadir levels.

Under SP, *Fbxl21* expression showed a complex waveform with a primary peak at ZT8-12 and a clear secondary peak around ZT0, giving an overall CoG of ZT8.48 ± 3.08. Following transfer to LP the principal mid-light phase peak became progressively delayed (LP3 CoG 9.95 ± 2.66; LP15 CoG ZT11.55 ± 2.84). Daily mean levels were increased by 40% and 80% in the LP3 and LP15 groups, respectively (p < 0.001 for overall effect of photoperiod). As for the *Per1-2* genes, this effect was due to an extension of the period when expression was above nadir levels, and to an increase in maximal levels of expression.

Contrasting to these effects for clock genes peaking in the light phase, the dark-expressed gene *Bmal1* responded less dramatically to the photoperiodic switch (p < 0.05 for Photoperiod x ZT interaction). Consistent with previous data [36], *Bmal1* expression peaked in the early to mid dark phase in SP acclimated sheep (CoG ZT14.32 ± 2.79), and declined to a pronounced nadir in the early light phase when expression levels were some 7-fold lower (p < 0.001 by one way ANOVA). This rhythmicity was dampened by the switch to LP, with no significant effect of ZT in either the LP3 or LP15 groups. There was no significant effect of photoperiod treatment on overall cycle mean levels of expression.

### Neuropeptide gene expression in the sheep SCN

**Fig 2:**
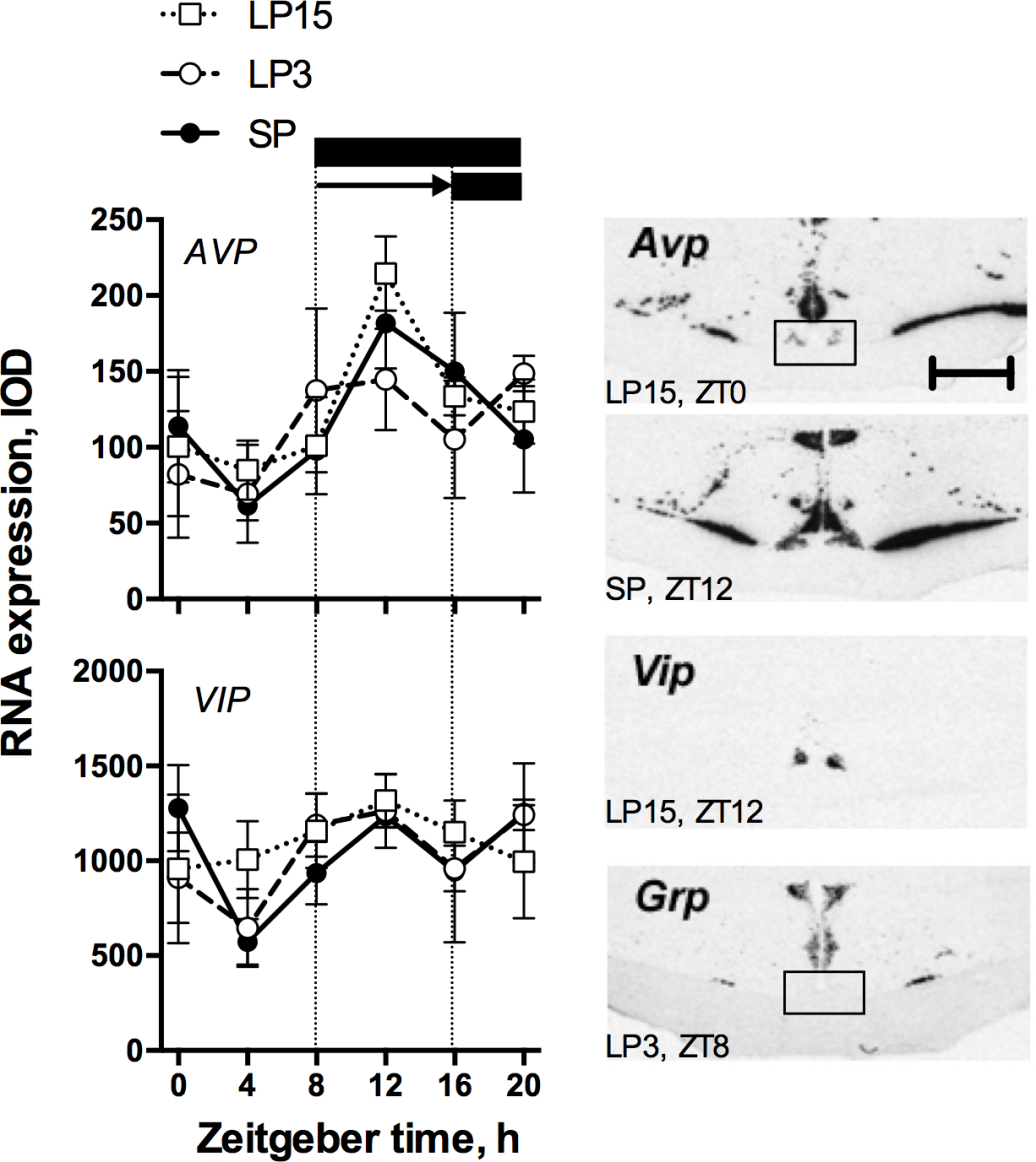
Neuropeptide gene expression in the sheep SCN. The left panel shows daily mRNA expression profiles for the neuropeptides *Avp* and *Vip*. *Avp* shows a pronounced daily rhythm of expression, but this was unaffected by the photoperiod manipulation. *Vip* expression shows no significant changes during the daily cycle, or in response to photoperiod manipulation. Data presentation in the graphs is as in Fig 1. The right panels show representative images for each of the 3 neuropeptide genes studied, with phases from which images were taken. Note the absence of *Grp* expression in the SCN despite strong labelling in the neighbouring supraoptic and paraventricular nuclei. Scale bar = 4 mm.

Representative images of autoradiograms for neuropeptide gene expression in the ovine SCN are shown in Fig 2.

The expression of *Avp* within the ovine SCN was dependent on time of day (p < 0.05 for effect of sampling time, ZT), but showed no significant change in waveform over the 15 days following the delay in lights off (photoperiod x ZT interaction, p = 0.93) (CoG estimates ranged from ZT14.49 ± 3.09 (SP) to ZT13.72 ± 3.1 (LP15)). In addition, no effect on overall cycle mean levels of expression was observed following the photoperiod switch (p = 0.78).

The expression of *Vip* was not affected by either time of day or photoperiod (two-way ANOVA; p = 0.78 and p = 0.87, respectively).

Finally, no labelling was found within the SCN for *Grp* in any of the animals, albeit high levels of expression were obvious in the hypothalamic supraoptic nuclei, paraventricular nuclei and the periventricular area.

## Discussion

Light synchronisation of the SCN circadian clock depends on direct retinal input. Due to their acute and phase-dependent light-responsiveness within the SCN [42–44], it is thought that *Per1* and *Per2* are key components of the photic entrainment pathway [45–47]. Light triggers glutamate and PACAP secretion within the SCN and both transmitters can evoke *Per1-2* gene induction and phase-shift the clock [48,49]. A classical intracellular signalling cascade involving cAMP and P-CREB appears to be responsible for the induction of *Per1-2* through cAMP Responsive Elements located within the promoter of these genes [50–52]. Our data are consistent with the view that *Per1* and *Per2* are light-inducible within the sheep SCN: The distribution of labelling for these genes overlaps with the retino-recipient zone of the sheep SCN as mapped by Tessoneaud et al [37], and both their level of expression and phase are rapidly shifted following the photoperiod extension. Indeed, when we compare the phases of peak period gene expression at LP3, with those reported in an earlier comparison of SP and LP acclimated Soay sheep [36], we can see that phase readjustment is already largely complete.

Additionally, in the present study we found that *Per1-2* gene expression profiles under LP3 and LP15 showed an increase in 24-h cycle mean levels of expression. This effect was not observed in the previous analysis of expression profiles in SP and LP acclimated Soay sheep [36]. Although we cannot exclude that this is a transient effect provoked by the photoperiod extension, it is also possible that the previous study in sheep underestimated effects on maximal levels of expression for technical reasons. We therefore favour the view that extended light exposure on the light-inducible elements of the SCN clockwork leads to heightened cycle mean levels of *Per1-2* gene expression. This explanation probably also accounts for the LP-induced doubling of 24-h cycle mean expression for *Fbxl21* observed in the present study.

In contrast to *Per1*/*Per2* and *Fbxl21*, exposure to LP blunted the peak to trough amplitude of the *Bmal1* daily rhythm. Such changes in daily amplitude of clock gene expression were not observed between SP-adapted vs LP-adapted sheep [36]. Indeed, although photoperiodic differences in phasing and duration of clock gene expression are consistently observed within the SCN of multiple rodents [31,32,53–56], no consistent systematic changes in the amplitude of RNA expression rhythms between SP and LP have emerged. Electrophysiological studies concur with this finding since photoperiod encoding has primarily been assigned to differences in the relative phase distribution between neurons, without modifications in the amplitude of rhythms in individual neurons [57–59].

*Avp* and *Vip* were both readily detected within the sheep SCN. The sheep differs from rodents, in that clear boundaries between core and shell SCN sub-compartments are not obvious, with *Avp* and *Vip* cells intermingled [37]. Closer examination of our autoradiograms confirmed this; although *Avp* and *Vip in-situ* hybridizations were performed on alternate sections it was not possible to assign expression to spatially distinct sub-regions. Investigation of retinal projections nevertheless suggests a certain level of compartmentalization [60].

AVP has been implicated as an output of the clock [5,61]. The mRNA profile of *Avp* is strongly rhythmic and the highest levels are found during the early night under SP and late light phase in LP3/LP15 groups. This rhythmic profile of expression is in broad agreement with what has been reported in many species, be they nocturnal or diurnal (references in [62]). This rhythm of *Avp* transcription within the SCN most likely accounts for the daily rhythm of vasopressin in cerebrospinal fluid reported in sheep [63], akin to what had been initially demonstrated by Schwartz and Reppert in rats [64]. But somewhat surprisingly the *Avp* rhythm was not modified in the course of the photoperiodic transfer. This contrasts with the situation in rodents, which typically show an increase in duration of *Avp* mRNA peak levels under LP compared to SP [32,55,56]. Since our previous studies [41,65] indicate that behavioural shifting occurs within the first week following a photoperiodic switch, we do not think the result reported here reflects incomplete re-entrainment at the SCN level. Rather, the reasons for this apparent difference may relate to the technical limitations of the radioactive *in situ* hybridisation approach, where clear spatial compartmentalisation of the SCN is lacking, or they may reflect differences in the functional role of AVP as a circadian SCN output in ungulates.

The mRNA profiles of *Vip* in the sheep SCN were neither rhythmic nor significantly different between SP and LP3/LP15. Large inter-specific differences in *Vip* expression and discrepancies in the effect of light have been reported: in rat, *Vip* mRNA and protein exhibit clear daily rhythms with high levels during the night and decreasing levels concomitant with lights-on (references in [62]; contrastingly, *Vip* mRNA is not rhythmic in the golden hamster [66], only a weak oscillation is observed in the Siberian hamster [67] and no variations of immunoreactivity are found within the human SCN [68]. Likewise, no large variations in mRNA levels are found in the mouse or the diurnal rodents *Arvicanthis ansorgei* and *Arvicanthis niloticus* exposed to a light-dark cycle [62,69]. These differences suggest that rhythmic *Vip* expression is not a prerequisite for SCN function across mammals.

Interestingly, we did not detect *Grp* mRNA within the ovine SCN. This might be relevant since *Grp*-expressing cells of the rodent SCN are the primary cell type in which *Per1* induction is observed after a light pulse, and are a likely gate for photic entrainment of the shell [12,14,69,70–74]. We cannot exclude the possibility that *Grp* is expressed within the sheep SCN, but at levels below the detection threshold of our *in situ* hybridization protocol. Indeed, the use of *in situ* hybridization to detect *Grp* expression within the SCN of rodents has consistently shown that levels are lower than those for *Avp* or *Vip* [12,75]. Another consistent observation across rodents is the limited rostro-caudal extent of *Grp* expression within the SCN, with neurons being found only in the mid-caudal SCN [12,14,69,71–74]. Therefore it might be that *Grp*-expressing neurons were not present in any of the sections we examined (3 sections per animal, total of 54 animals), because slides were taken in regions too rostral. However, considering that (i) we detected *Vip* in all sections and (ii) *Vip* and *Grp* expressing cells overlap in rodents, with some cells actually co-expressing both neuropeptides [14], we do not think this is the most likely explanation. Bearing in mind these plausible limitations, inherent to our technical approach, strong *Grp* expression was obvious in the supra-optic and paraventricular nuclei and in periventricular regions, areas devoid of *Grp* labeling in rodents to the best of our knowledge. Therefore, it would seem that GRP may play distinct roles in the hypothalamus of sheep and rodents.

In conclusion, the present study indicates that phase-shifting of the sheep SCN molecular clock during a long-day transfer is achieved rapidly, over the first 15 days of LP exposure. We notice that cycle mean levels of expression were increased in the course of the LP transfer for *Per1*, *Per2* and *Fbxl21* due to effects on peak expression levels, and on duration of the period when expression was above nadir levels. This was not seen for *Bmal1*, indicating that this effect is gene-specific. We found that although expression of *Avp* was rhythmic, photoperiodic transfer did not lead to an overt change in phase. Finally, the expression of *Vip* was not rhythmic in any of the three groups and *Grp* does not appear to be expressed in the SCN of the sheep. Overall our data suggest that the mechanisms of photoperiodic synchronisation at the SCN level are broadly similar between ungulates and rodents, with however some differences in neuropeptide signalling.
